# Association between Ophthalmic Timolol and Hospitalisation for Bradycardia

**DOI:** 10.1155/2015/567387

**Published:** 2015-03-22

**Authors:** Nicole L. Pratt, Emmae N. Ramsay, Lisa M. Kalisch Ellett, Tuan A. Nguyen, Elizabeth E. Roughead

**Affiliations:** Quality Use of Medicines and Pharmacy Research Centre, School of Pharmacy and Medical Sciences, University of South Australia, Adelaide, SA 5001, Australia

## Abstract

*Introduction*. Ophthalmic timolol, a topical nonselective beta-blocker, has the potential to be absorbed systemically which may cause adverse cardiovascular effects. This study was conducted to determine whether initiation of ophthalmic timolol was associated with an increased risk of hospitalisation for bradycardia. *Materials and Methods*. A self-controlled case-series study was undertaken in patients who were hospitalised for bradycardia and were exposed to timolol. Person-time after timolol initiation was partitioned into risk periods: 1–30 days, 31–180 days, and >180 days. A 30-day risk period prior to initiating timolol was also included. All remaining time was considered unexposed. *Results*. There were 6,373 patients with at least one hospitalisation for bradycardia during the study period; 267 were exposed to timolol. Risk of bradycardia was significantly increased in the 31–180 days after timolol initiation (incidence rate ratio (IRR) = 1.93; 95% confidence interval (CI) 1.00–1.87). No increased risk was observed in the first 30 days or beyond 180 days of continuous exposure (IRR = 1.40; 95% CI 0.87–2.26 and IRR = 1.21; 95% CI 0.64–2.31, resp.). *Conclusion*. Bradycardia is a potential adverse event following timolol initiation. Practitioners should consider patient history before choosing a glaucoma regime and closely monitor patients after treatment initiation with topical nonselective beta-blocker eye drops.

## 1. Introduction

The prevalence of glaucoma, a leading cause of vision loss [1], increases with age [2]. The number of people with glaucoma in Australia is predicted to increase from 208,000 in 2005 to 379,000 in 2025 [3]. The most common form of medical management of glaucoma is the use of topical eye drops that reduce intraocular pressure [4]. *β*-adrenergic antagonists (*β*-blockers) are the most commonly prescribed glaucoma medicines in a number of countries including the United Kingdom [5] and the United States [6]. In Australia, treatment options include prostaglandin analogues, which are now the most common treatment for glaucoma [4]. *β*-blockers still have substantial usage [4] and many combination products are available which contribute to their use.

Timolol is a potent nonselective *β*-blocker and was the mainstay of glaucoma therapy through the 1980s and 1990s [2, 7] because it is effective in lowering intraocular pressure [8], associated with few ocular side effects and does not affect pupil size [9, 10]. Although administered topically, when used in the eye timolol can reach the systemic circulation through the nasolacrimal duct, the conjunctival vessels, and gastrointestinal tract [11–13]. The systemic bioavailability and pharmacokinetics of ophthalmic timolol 0.5% are comparable to intravenous timolol [13]. A dose of one drop of 0.5% timolol solution to each eye is equivalent to a 10 mg oral dose [14, 15]. Systemic adrenergic *β*-blocking effects of ophthalmic timolol may therefore occur, including effects on cardiac, pulmonary, central nervous system, and endocrine functions [6, 16].

Change in heart rate is one of the effects of the systemically absorbed fraction of ophthalmic timolol [6, 17]. In the first seven years of commercial production of ophthalmic timolol in the United States, 450 serious adverse cardiopulmonary events were reported, and 32 deaths were attributed to the use of ophthalmic timolol. Preexisting cardiovascular disease was reported in 31% of the 212 persons for whom medical history was provided [18]. Ophthalmic timolol is therefore contraindicated in patients with certain cardiovascular disorders, including bradyarrhythmias and atrioventricular block [2].

A number of studies have been conducted to confirm the systemic *β*-blocking effects of ophthalmic timolol on cardiovascular functions, including bradycardia, and results varied substantially across studies. Randomised controlled trials and crossover studies found a range in reduction of resting heart rate and in peak heart rate during exercise, from negligible to an 11 beat per minute (bpm) reduction and from 5 to 22 bpm reduction, respectively, depending on the types of ophthalmic timolol: 0.25–0.5% aqueous or 0.1–0.5% hydrogel formulations [13, 19–33].

Given these findings, we aimed to quantify the potential risks of hospitalisation for bradycardia following initiation of ophthalmic timolol in an elderly population.

## 2. Materials and Methods

### 2.1. Data Source

The Australian Government Department of Veterans' Affairs (DVA) administrative claims database was used in this study. Details of all prescription medicines, medical and allied health services, and hospitalisations for which DVA pays a subsidy are available. Data are available for a treatment population that in September 2011 was 242,147 people [34] and who had a median age of 80 years. DVA maintains a client file, which includes data on gender, date of birth, date of death, and family status. Medicines are coded in the dataset according to the World Health Organization (WHO) Anatomical Therapeutic Chemical (ATC) Classification [35] and the Schedule of Pharmaceutical Benefits Item Codes [36]. Hospitalisations are coded according to the International Classification of Diseases, version 10, Australian modification (ICD-10-AM) [37].

### 2.2. Study Design

The self-controlled case-series design [38, 39], which is a within person design, was used to compare the rate of hospitalisation for bradycardia during periods of exposure to timolol compared to unexposed periods. Eligible persons were those who were hospitalised for bradycardia (primary diagnosis ICD-10AM R001, I440, I441, I442, I443, and I495) between July 1, 2003, and June 30, 2009. Persons were included if they were aged 65 years or over at the start of the study, eligible for all health services subsidised by DVA and were dispensed at least one medicine in the year prior to the start of the study. To focus this analysis on new users of ophthalmic timolol, subjects who were dispensed ophthalmic timolol (ATC code S01ED01) or combination medicines with timolol (ATC code S01ED51) in the year prior to the start of the study were excluded. Subjects were followed until death or the end of the study period (June 30, 2009), whichever occurred first.

### 2.3. Statistical Analysis

As timolol eye drops are required to be discarded four weeks after opening, exposure duration for each dispensed ophthalmic timolol was defined as 35 days, allowing for an additional week to account for late prescription refill. Patients with repeat dispensings within 35 days were considered to be continuously exposed. The end of the exposure period was defined as 35 days after the last dispensing of timolol eye drops where no subsequent dispensing occurred. For those patients who had at least one timolol dispensing during the study period, their exposed time was partitioned into the following risk periods: 1–30 days, 31–180 days, and all remaining exposure time after timolol initiation (>180 days). A preexposure risk period of 30 days prior to initiating treatment with timolol was included to ensure the occurrence of the outcome was not altering the probability of subsequent exposure, a fundamental assumption of the self-controlled case series method. The actual day of prescription was excluded from this analysis as we were unable to define the temporal association between the exposure and a hospitalisation if they occurred on the same day. The incidence of outcomes in each of the exposure risk periods was compared to the incidence of outcomes in the unexposed reference period. Incidence rate ratios (IRRs) were calculated using conditional poisson regression adjusting for age at hospitalisation and calendar year.

Sensitivity analyses were performed by including additional adjustments for covariates including concomitant prescribing of oral beta-blockers (ATC code C07), calcium-channel blockers (ATC code C08), digitalis glycosides (ATC code C01AA), and antiarrhythmics (ATC code C01B). We also included patients who were hospitalised for bradycardia but not exposed to timolol during the study period to adjust for the possibility of increasing incidence of bradycardia hospitalisation with age. These patients contributed information on the impact of time varying covariates, including age, on the risk of the outcome [38, 39]. All person-time for the unexposed group was included in the unexposed reference period. We also stratified by concomitant use of oral beta-blockers. All analyses were performed using SAS version 9.12 (SAS Institute, Cary, NC).

## 3. Results

The demographics of the study population are presented in Table 1. There were 6,373 veterans with at least one hospitalisation for bradycardia during the study period, with 267 exposed to timolol and 6,106 never exposed. Of the study population, 59.6% were males. The mean age at first hospitalisation was 82.6 years.

There was no statistically significant increase in the risk of hospitalisation for bradycardia in the first 30 days after initiating timolol (incidence rate ratio (IRR) = 1.40; 95% confidence interval (CI) 0.87–2.26). The risk of bradycardia was significantly increased in the 31 to 180 days after timolol initiation (IRR = 1.93; 95% CI 1.30–2.87), but did not remain statistically significantly elevated thereafter (Table 2).

Results were similar after adjusting for other conditions and when including unexposed patients (Table 3). The stratified analyses also show similar risk estimates for patients not taking oral beta-blockers; however, the risk in the 31–180-day risk period was not statistically significant (IRR = 1.76; 95% CI 0.88–3.50) (Table 3).

## 4. Discussion 

Timolol is a nonselective *β*-blocker; thus it is a risk factor for cardiovascular functions. Most of the published evidence of the systemic *β*-blocking effects of ophthalmic timolol on cardiovascular functions was from randomised controlled trials (RCTs), cross-over studies, or case reports [13, 18–33]. Participants included in RCTs were often very selective (e.g., healthy people) and not representative of the real-world population. RCTs excluded the elderly in whom *β*-blockade has been found to be stronger and last longer. In addition, RCTs were limited to investigating the impact of timolol on resting heart rate and peak heart rate during exercise; however, the impact of timolol on the more serious outcome of hospitalisation for bradycardia was not assessed.

Using the DVA administrative claims database in this observational study, we found an increased risk of hospitalisation for bradycardia one month after initiation of timolol eye drops. The increased risk of hospitalisation for bradycardia was reduced and no longer statistically significant after six months of continuous treatment. One explanation for this finding may be that those patients who continue to take ophthalmic timolol for extended periods are those with better tolerance, thus being less likely to experience the adverse event. Our findings are in line with previous clinical trial evidence and case reports which suggested that ophthalmic timolol was associated with adverse cardiac effects [18, 40].

Despite evidence linking the use of topical *β*-blockers to bradycardia, codispensing of ophthalmic *β*-blockers with medicines which can cause or exacerbate bradycardia is common. We previously showed that 36% of those with glaucoma who were dispensed verapamil were also codispensed ophthalmic timolol [41], a contraindication which may worsen bradycardia [42]. Interventions raising awareness of these potential adverse events with prescribers are required. The majority of glaucoma medicines are initiated by ophthalmologists, while adverse events may be managed by general practitioners, raising challenges of how to address adverse events across the continuum of care. Cross-specialty cooperation is therefore needed to optimise patient care with improved communication among ophthalmologists, general practitioners, pharmacists, and patients regarding the history of cardiac diseases and glaucoma treatment.

The use of the self-controlled case series design where the patient implicitly acts as their own control adjusts for all confounders that remain fixed over the observation period, including sex, location, genetics, and underlying state of health [38]. The absence of diagnostic information in the DVA dataset means that disease severity could not be taken into account. However, sensitivity analyses were performed by adjusting for concurrent medications uses, which are the proxy for the presence of conditions that may impact on the risk of hospitalisation for bradycardia. These analyses made little difference to the risk estimates suggesting that the increased risk of hospitalisation for bradycardia is unlikely to be due to confounding because of changes in disease severity.

In Australia, ophthalmic timolol is registered for ocular hypertension and glaucoma, and the DVA dataset does not allow distinguishing which condition the exposed individuals had. In addition, dosage information is not available in the dataset, so we were unable to assess the dose-response relationship. The selection of the veteran population in this study may be seen as another limitation for the generalisation of our findings. However, previous research has shown that there was no difference in use of practitioners, health services, and pharmaceuticals between war veterans and nonveteran patients in both the primary and tertiary Australian care sectors after adjustment for age, service-related disability, and marital status [43]. Our results, which have consolidated scientific evidence on the risk of hospitalisation for bradycardia, are therefore likely to be applicable to the elderly Australian population and suggest that this adverse event is still occurring.

## 5. Conclusion

Bradycardia is a potential adverse event following timolol initiation. Practitioners should be reminded to carefully examine the patient history before choosing a glaucoma regime and closely monitor patients after treatment initiation with topical nonselective beta-blocker eye drops to minimise adverse events and potentially avoid hospitalisations.

## Figures and Tables

**Table 1:** Demographics of the study cohort.

Demographics	Bradycardia hospitalisation cohort (*n* = 6,373)		
	Exposed (*n* = 267)	Never exposed (*n* = 6,106)	Whole cohort (*n* = 6,373)
Age, mean (SD), year	82.7 (4.8)	82.6 (4.8)	82.6 (4.8)
Male gender, No. (%)	154 (57.7)	3645 (59.7)	3799 (59.6)
Number of medicines used, median (IQR)^a^	12 (8–17)	12 (8–18)	12 (8–18)
Number of prescribers, median (IQR)^a^	12 (8–16)	12 (7–17)	12 (7–17)
Number of specialist visits, median (IQR)^a^	5 (2–8)	3 (1–8)	3 (1–8)
Number of hospitalisations, median (IQR)^a^	1 (0–2)	1 (0–2)	1 (0–2)
Number of comorbidities, median (IQR)^b^	5 (4–7)	5 (3–7)	5 (3–7)

SD: standard deviation; IQR: interquartile range; and No.: number.

^a^Values for 12 months prior to study entry.

^b^Value for time-varying every 4 months.

**Table 2:** Self-controlled case series results for patients with a hospitalisation for bradycardia and at least one dispensing of timolol.

Risk periods	*N* hospitalisations	Person-years	Adjusted rate^a^ per 10 years (95% CI)	IRR^a^ (95% CI)
Unexposed	161	1112	1.42 (1.17–1.71)	1.00 (1.00-1.00)
Before 1–30 days	2	22	0.82 (0.20–3.30)	0.58 (0.14–2.35)
After 1–30 days	25	127	1.98 (1.30–3.03)	1.40 (0.87–2.26)
After 31–180 days	58	234	2.73 (1.99–3.75)	1.93 (1.30–2.87)
After 180 days	17	121	1.72 (0.96–3.07)	1.21 (0.64–2.31)
Washout	4	63	0.60 (0.22–1.63)	0.42 (0.15–1.17)

CI: confidence interval; IRR: incidence rate ratio.

^a^Adjusted for age at hospitalisation and calendar year.

**Table 3:** Sensitivity analyses.

Risk periods	*N* hospitalisations	Person-years	Adjusted rate per 10 years (95% CI)	IRR (95% CI)
Exposed patients only (adjusting for age, calendar year, and other conditions^a^)				
Unexposed	161	1112	1.45 (1.20–1.75)	1.00 (1.00-1.00)
Before 1–30 days	2	22	0.86 (0.21–3.46)	0.59 (0.15–2.41)
After 1–30 days	25	127	2.02 (1.32–3.10)	1.40 (0.86–2.26)
After 31–180 days	58	234	2.78 (2.02–3.81)	1.91 (1.28–2.85)
After 180 days	17	121	1.75 (0.98–3.13)	1.21 (0.63–2.30)
Washout	4	63	0.62 (0.23–1.69)	0.43 (0.16–1.19)

Including unexposed patients (adjusting for age and calendar year)				
Unexposed	6267	36791	1.39 (1.33–1.46)	1.00 (1.00-1.00)
Before 1–30 days	2	22	0.74 (0.18–3.00)	0.53 (0.13–2.16)
After 1–30 days	25	127	1.77 (1.11–2.82)	1.27 (0.80–2.02)
After 31–180 days	58	234	2.47 (1.70–3.59)	1.77 (1.22–2.58)
After 180 days	17	121	1.58 (0.85–2.93)	1.13 (0.61–2.10)
Washout	4	63	0.54 (0.20–1.48)	0.39 (0.14–1.06)

Exposed patients only who were dispended at least one oral beta-blocker (adjusting for age and calendar year)				
Unexposed	97	690	1.43 (1.11–1.86)	1.00 (1.00-1.00)
Before 1–30 days	2	13	1.36 (0.34–5.46)	0.95 (0.23–3.88)
After 1–30 days	12	81	1.54 (0.85–2.80)	1.08 (0.55–2.09)
After 31–180 days	40	155	2.95 (2.03–4.31)	2.06 (1.26–3.36)
After 180 days	12	76	2.10 (1.06–4.15)	1.46 (0.68–3.15)
Washout	3	39	0.77 (0.24–2.43)	0.54 (0.16–1.75)

Exposed patients only who were not dispended oral beta-blockers (adjusting for age and calendar year)				
Unexposed	64	422	1.51 (1.11–2.05)	1.00 (1.00-1.00)
Before 1–30 days	0	88	—	0.95 (0.23–3.88)
After 1–30 days	13	46	3.04 (1.61–5.73)	2.01 (0.99–4.08)
After 31–180 days	18	79	2.65 (1.46–4.80)	1.76 (0.88–3.50)
After 180 days	5	45	1.18 (0.39–3.55)	0.78 (0.24–2.55)
Washout	1	24	0.42 (0.06–3.05)	0.28 (0.04–2.06)

CI: confidence interval; IRR: incidence rate ratio.

^a^Oral beta-blockers (ATC code C07), calcium-channel blockers (ATC code C08), digitalis glycosides (ATC code C01AA), and antiarrhythmics (ATC code C01B).
